# FIRE (facilitating implementation of research evidence): a study protocol

**DOI:** 10.1186/1748-5908-7-25

**Published:** 2012-03-27

**Authors:** Kate Seers, Karen Cox, Nicola J Crichton, Rhiannon Tudor Edwards, Ann Catrine Eldh, Carole A Estabrooks, Gill Harvey, Claire Hawkes, Alison Kitson, Pat Linck, Geraldine McCarthy, Brendan McCormack, Carole Mockford, Jo Rycroft-Malone, Angie Titchen, Lars Wallin

**Affiliations:** 1 Royal College of Nursing Research Institute, School of Health and Social Studies, University of Warwick, Coventry CV4 7AL, UK; 2 Fontys University of Applied Sciences School of Nursing, PO Box 347, 5600, AH Eindhoven, the Netherlands; 3 Faculty of Health and Social Care, London South Bank University, 103 Borough Road, London SE1 0AA, UK; 4 Bangor University, Centre for Economics and Policy in Health/Canolfan Economeg a Pholisi Iechyd, IMSCaR, College of Health and Behavioural Sciences, Dean Street Building, Bangor University, Bangor LL57 1UT, UK; 5 Department of Neurobiology, Care Sciences and Society, Division of Nursing, Karolinska Institutet and Clinical Research Utilization (CRU), Karolinska University Hospital, Eugeniahemmet T4:02, SE-171 76 Stockholm, Sweden; 6 Faculty of Nursing, University of Alberta, Edmonton, Alberta T6G 2G3, Canada; 7 Health Management Group, Manchester Business School, University of Manchester, Manchester M15 6PB, UK; 8 Bangor University, Centre for Health Related Research, School of Healthcare Sciences, College of Health and Behavioural Sciences, Fron Heulog, Bangor University, Bangor, Gwynedd LL57 2EF, UK; 9 School of Nursing, University of Adelaide, Adelaide, 5005, Australia; 10 University College Cork, College of Medicine & Health, Cork, Republic of Ireland; 11 Institute of Nursing Research/School of Nursing, University of Ulster, Newtownabbey, BT37 0QB, Northern Ireland

## Abstract

**Background:**

Research evidence underpins best practice, but is not always used in healthcare. The Promoting Action on Research Implementation in Health Services (PARIHS) framework suggests that the nature of evidence, the context in which it is used, and whether those trying to use evidence are helped (or facilitated) affect the use of evidence. Urinary incontinence has a major effect on quality of life of older people, has a high prevalence, and is a key priority within European health and social care policy. Improving continence care has the potential to improve the quality of life for older people and reduce the costs associated with providing incontinence aids.

**Objectives:**

This study aims to advance understanding about the contribution facilitation can make to implementing research findings into practice via: extending current knowledge of facilitation as a process for translating research evidence into practice; evaluating the feasibility, effectiveness, and cost-effectiveness of two different models of facilitation in promoting the uptake of research evidence on continence management; assessing the impact of contextual factors on the processes and outcomes of implementation; and implementing a pro-active knowledge transfer and dissemination strategy to diffuse study findings to a wide policy and practice community.

**Setting and sample:**

Four European countries, each with six long-term nursing care sites (total 24 sites) for people aged 60 years and over with documented urinary incontinence

**Methods and design:**

Pragmatic randomised controlled trial with three arms (standard dissemination and two different programmes of facilitation), with embedded process and economic evaluation. The primary outcome is compliance with the continence recommendations. Secondary outcomes include proportion of residents with incontinence, incidence of incontinence-related dermatitis, urinary tract infections, and quality of life. Outcomes are assessed at baseline, then at 6, 12, 18, and 24 months after the start of the facilitation interventions. Detailed contextual and process data are collected throughout, using interviews with staff, residents and next of kin, observations, assessment of context using the Alberta Context Tool, and documentary evidence. A realistic evaluation framework is used to develop explanatory theory about what works for whom in what circumstances.

**Trial registration:**

Current Controlled Trials ISRCTN11598502.

## Background

Evidence-based healthcare has featured as a policy concern in many healthcare systems for over a decade driven by a growing recognition and concern that healthcare practice does not always reflect what is known to be best practice. Some studies [1,2] suggest that twenty to forty per cent of patients receive harmful care or care that is inconsistent with scientific evidence. Responding to these concerns, policy makers have increasingly sought ways to narrow the research-practice gap and ensure that research is translated into clinical practice as effectively and efficiently as possible. Initiatives have included the establishment of national guideline development and technology assessment bodies [3,4] knowledge and skills development of healthcare staff [5,6], and research programmes to investigate effective implementation methods and processes [7,8].

Despite significant investment, translating research in to healthcare decision making and practice remains a considerable challenge. In the United Kingdom (UK), a national evaluation of the extent and pattern of implementation of guidance issued by the National Institute for Health and Clinical Excellence (NICE) demonstrated a highly variable level of uptake, ranging from no change to significant changes in practice in line with the guidance [9]. Additionally the literature contains numerous examples of attempts to implement evidence into practice; with mixed success [10-14]

Translating and using research in practice is complex, involving significant and planned individual, team, and organisational change. A case study meta analysis of evidence into practice projects highlights the social, organisational, and professional factors that mediate evidence use [15] and are consistent with a review of the diffusion of innovations literature, which emphasised the complex interactions between clinicians and their practice settings [16], illustrating that there are no simple solutions to complex healthcare problems.

The complexities inherent in the implementation of evidence into practice are represented in the Promoting Action on Research Implementation in Health Services (PARIHS) framework [17-21]. In contrast to previous frameworks that had represented implementation as a linear and rationale process, PARIHS was developed to demonstrate the complex interplay of a number of factors that influence the successful implementation of evidence in practice [17]. Successful implementation is represented as a function of the nature of evidence being implemented, the context in which implementation takes place, and the way in which that process is facilitated: SI = f(e, c, f).

The subsequent development and refinement of the framework has been comprehensively described in recent publications [19,20]. This development has included concept analyses of the core concepts within the framework: evidence [22], context [23], and facilitation [24]. As a result, the framework has evolved to provide a map to enable others to make sense of the complexities of implementation and the elements that require attention if implementation is more likely to be successful [21].

The PARIHS framework has been well received by those working in the field of evidence-based healthcare, and has been used by others as a heuristic to guide implementation efforts at the point of care delivery [25-27] and as the conceptual underpinning of a variety of tools and measures [28,29]. A critical synthesis of empirical studies in which the PARIHS framework was used highlighted its strengths and issues. Their conclusions included the need for further delineation of the elements within the framework, and a call for the framework to be used prospectively in implementation studies [30]. The FIRE study is one attempt to do this, with a particular focus on the facilitation dimension of the PARIHS framework.

A key element of the PARIHS framework is facilitation, which could be described as a mechanism or intervention for the implementation of evidence into practice. A facilitator is an individual who is skilled in working with the concepts of change management and individual and organisational development. Facilitation involves the facilitator working with individuals, teams, and organisations to prepare, guide, and support them through the implementation process. This involves attentiveness to both the context (including barriers and enablers of change) and to the evidence to be implemented and how it fits with local circumstances. Two important features were identified in a concept analysis of facilitation [24]. Firstly, not all efforts to get research evidence into practice explicitly engage processes to support implementation. In such cases, implementation involved discrete interventions, such as the distribution of printed materials or the provision of targeted educational meetings, the assumption being that on learning about the new evidence, practitioners would change their practice accordingly. Secondly, in cases where interventions to promote implementation did involve an individual taking on a facilitator role (for example, a dedicated project lead, educational outreach worker, or practice development facilitator), two models of facilitation were apparent. These different models were represented along a continuum, ranging from a largely task-focused, project manager role, to a more holistic, enabling approach to facilitation where the facilitator worked at the level of individuals, teams, and organisations to create and sustain a supportive context for evidence based care [31,32].

The findings from the concept analysis of facilitation suggest that the key to successful implementation is matching the purpose, role, and skills of the facilitator to the specific needs of the situation, i.e., appropriate facilitation [24]. A systematic review of 26 studies concluded that whilst tailored interventions can change practice, there is a lack of evidence about how interventions should be selected to address barriers, and no evidence on cost effectiveness [33]. Given the increasingly recognised complex nature of implementing research evidence into practice and the need to address the interplay between individuals and the organisation [15,34], it is reasonable to suggest that skilled facilitators need to be able to move across different points of the facilitation continuum to meet the different requirements of individuals, teams, and organisations at different points in time. However, this requires facilitators to possess a sophisticated range of knowledge and skills, including, for example, diagnostic skills (to assess the organisational context and the needs of individuals and teams within that context), project management skills, and a range of skills to support individual, team, and organisational development and learning. In turn, this requires significant investment of resources and time, both in preparing and supporting individuals to take on the facilitator role and in creating time for individuals and teams to work with the facilitator to implement research evidence into practice. Other issues to consider include the need to distinguish between the facilitator role and the methods that the facilitator uses, the complexity of the role and how much facilitation is needed in a given situation, and the need to better understand the relationship between facilitation, context, and evidence.

Despite the increasing use of facilitation type models and techniques within implementation projects, to date, facilitation has received little attention in the formal classification of methods and interventions to change professional and organisational behaviour (for example, by the Cochrane review group on Effective Practice and Organisation of Care). Systematic reviews of various implementation interventions across a variety of settings show mixed effects [35-37]. There is some evidence to support the use of discrete interventions such as the distribution of printed materials, the use of reminders, audit and feedback, participative education programmes, and social marketing techniques. However, further research is needed into the effectiveness of such implementation interventions, and more specifically there has been a call for more theory informed interventions, and robust and methodologically sound research [36,38,39].

This protocol describes a theory driven study to evaluate the effectiveness of facilitation as an intervention to implement evidence into practice. Drawing on the core elements of evidence, context, and facilitation, we will test a number of theoretical propositions about facilitation and the implementation of continence promotion evidence within nursing home settings across four European countries. The research will start to uncover the relationship between particular facilitation methods and the impact of the use of these methods on evidence use, contextual change, team effectiveness, and the creation of organisational infrastructures that support and enable knowledge utilisation.

## Methods

### Aims

1. Extend current knowledge of facilitation as a process for translating research evidence into practice. Based on the PARIHS framework, two different models of facilitation will be developed (described as technical [Type A] and enabling [Type B] facilitation), requiring different levels of facilitator skills and knowledge and the application of different methods of implementation, with corresponding different levels of resource requirements in terms of preparation and support of facilitators and the ways in which they work with individuals and teams who are attempting to implement research into practice.

2. Evaluate the feasibility, effectiveness, and cost-effectiveness of two different models of facilitation in promoting the uptake of research evidence on continence management. An intervention study in four countries (England, Sweden, Republic of Ireland, Netherlands) will be set up to test the two different models of facilitation against a standard method of disseminating evidence of best practice on continence promotion. Six units per country will participate in the study (two units for each of the three study arms). The research evidence to be implemented will draw on existing evidence in the form of systematic reviews and guidance on continence management.

3. Assess the impact of contextual factors on the processes and outcomes of implementation. The research will be underpinned by a theory-driven methodology, with a particular focus on explaining what works, for whom, how and in what circumstances. A detailed set of contextual, process, and outcome data will be collected in all the study sites to track the processes of implementation, to account for and explain contextual differences between and within countries, to monitor changes over time, and any diffusion beyond each study setting.

4. Implementing a pro-active knowledge transfer and dissemination strategy to diffusion of the study findings to a wide policy and practice community. Dissemination will be planned in parallel to the design and implementation of the study, reflecting the theory-driven nature of the research and the importance of stakeholder involvement at all stages of the research process. Stakeholders will include the commissioners of the research, key interest groups at a European level, and policy-makers, managers, and practitioners in EU countries not actively participating in the research. Stakeholder involvement will inform the development and refinement of theoretical propositions as the study progresses and findings begin to emerge. A range of networking and dissemination methods will be used to promote the input and involvement of countries from throughout Europe and beyond.

### Overall design

The design of this study is a pragmatic randomised controlled trial (RCT) with integral qualitative, quantitative and health economic evaluative components. The RCT has three arms: standard dissemination of recommendations; standard dissemination plus Type A (technical) facilitation; and standard dissemination plus Type B (enabling) facilitation.

We will assess the impact of the different facilitation programmes on processes and outcomes when implementing continence recommendations in long-term nursing care settings for older people. A dissemination or knowledge transfer strategy runs concurrently with the rest of the study.

Whilst researchers investigating knowledge translation routinely collect summative data in RCTs of interventions, they rarely collect process data (Grimshaw et al. 2004). Critical questions about the context of implementation, the feasibility of interventions, participant response, and resource implications need to be addressed in order to appraise why interventions worked (or not), in what situations, and with which stakeholders. This study has been designed to ensure that the experimental design is combined with exploratory and explanatory research in order to develop a richer and more detailed picture of the process and impact of implementation. As such, the findings of the study will have the potential to significantly build on and contribute to the international evidence base about research implementation. This study enables us to explore the factors that affect implementation over time within sites. The process evaluation has been designed as a realistic evaluation, and is embedded into the trial design. Realistic evaluation [40] is particularly relevant for this work because it aims to develop explanatory theory about why interventions and strategies work, for whom, how, and in what circumstances. This will allow a detailed understanding of how the processes of facilitation relate to outcomes. This multi-level evaluation will be theory-driven and informed by key concepts within the programme's theoretical framework, and by knowledge translation theory more broadly. The economic evaluation is fully integrated into the evaluation and will consider both the costs and consequences of the two facilitation models compared to standard dissemination of the continence recommendations.

### Inclusion Criteria

#### Setting

Long-term nursing care settings with publically funded places, at least 60 residents, are interested in taking part, have residents who are aged 60 years or older, with documented urinary incontinence. Settings will be excluded if they have previously taken part in other externally led improvement projects and/or where staff have already had facilitation development.

#### Participants

##### Staff

Staff who have consented to be involved as: internal facilitators engaged in intervention delivery; staff at all levels working in sites delivering care; and key stakeholders related to sites (e.g., community based nurse).

##### Residents

All residents meeting the following criteria are eligible for the study: aged 60 or over with no or mild dementia who consent to access to their continence related notes and/or being observed and/or completing quality of life questionnaires and/or taking part in interviews. Next of kin or consultee will be asked about looking at continence-related notes of residents with moderate to severe dementia. (A consultee is a person who, as a result of an existing relationship with the person who lacks capacity, can advise the researcher about that person's participation in the project).

##### Informal carers

Informal carers such as next of kin of residents who consent to be interviewed are eligible.

##### Exclusion criteria

Residents with moderate to severe dementia will not be interviewed or asked to complete quality of life scales. However, advice from next of kin or consultee will be sought about looking at their nursing home notes about continence care. In addition, next of kin or consultee of residents with moderate to severe dementia will be asked to completed a proxy quality of life scale (EQ-5D).

##### Facilitation intervention

The participants on the facilitation development programmes, known as internal facilitators, will be members of staff in the organisations in which the long-term care settings are located. They will be staff who are usually asked by their managers to lead and/or facilitate projects, change, or innovate in that organisation. Payment at a specified level will be made by the study to meet the costs of covering shifts while the internal facilitators take part in the study. The facilitation programmes will be led by expert external facilitators from the study team, working with the internal facilitators. To manage the risk associated with an internal facilitator leaving during the study, a co-facilitation model will be used, where a second person works with the internal facilitator, using this as a development opportunity, including taking the lead if the initial facilitator is unable to continue. This has been used successfully in other settings by the external facilitators [41].

Two facilitation programmes were developed (type A and type B described below) to help participants implement recommendations for continence care in long-term nursing care settings for older people. These two types of facilitation intervention were informed by relevant theoretical and empirical knowledge, and facilitated by international experts in the area (the external facilitators).

##### Arm One -- Control Group

The control group will receive standard dissemination that involves distributing the recommendations for continence care to the head of the long-term care setting, with a PowerPoint presentation about implementation. This is seen as standard practice (RCN 2007).

##### Arm Two -- Type A facilitation plus standard dissemination

This technical facilitation focuses particularly on addressing issues of implementation at the level of clinical teams, in terms of enabling them to design systems and processes of care that will enhance the transfer of evidence into their day-to-day practice. Type A facilitation draws on an eclectic range of theories, derived from management science, organisational learning, quality improvement, and humanistic psychology. It adopts a pragmatic approach to implementation, whilst recognising the need to pay attention to task, group, and individual needs. Individuals are prepared to take on the role of facilitator and are provided with a 'toolkit' of methods and techniques that they can use with health care teams to facilitate both the task and the process of implementing evidence [41]. This is a 12-month development programme. Internal facilitators will have 19 days of protected time over the year (this will consist of 10 days to work on the implementation and evaluation of the clinical guidelines; three days residential training; 12 half days for monthly telecommunications support by external facilitators and self-directed study). External facilitators will require 16 days each over the 12 months to fulfil their facilitation role. The external facilitators for Type A facilitation are members of the study team (GH and AK).

##### Arm Three -- Type B facilitation plus standard dissemination

This enabling facilitation uses critical social science concepts (e.g., consciousness-raising, problematisation, self-reflection, and critique) on the basis that the emphasis on inquiry and the development of individual practitioners, cultures, and contexts within which they work, will result in overcoming difficulties, through emancipatory action, and thus bring about sustainable change. Such action is collaborative, inclusive, and participative. Theoretical underpinnings also draw on critical creativity and its focus on creating conditions for person-centredness and human flourishing and engaging in praxis (mindful, practical action) through professional artistry. Enabling facilitation is concerned with change and innovation, individual and team effectiveness, leadership and evidence use and development. Recent evidence suggests that practice development achieved through enabling facilitation methods requires a minimum of two years of sustained enabling facilitation [42]. Arm three, therefore, is a two-year, two-staged intervention.

In stage one, the aim is to provide an opportunity for internal facilitators to develop, use, and refine a practitioner inquiry approach to enabling stakeholders in their organisations to improve continence care of residents by getting research into practice. This comprises the delivery of an 18-month enabling facilitation development programme, including planning, implementation, and evaluation of the continence recommendations. Stage two is a six-month diffusion study with the aim of enabling internal facilitators to develop further person-centred, evidence-informed practice in their workplaces/organisations. Internal facilitators will have 43 protected days over the two years (20 days to work on the implementation, evaluation and diffusion of clinical guidelines, five days residential learning, 24 half day learning groups supported via telecommunications by the external facilitators), and 12 half days for self-directed study and research, culminating in the production of a portfolio of evidence. External facilitators will require 31 days each over two years. The external facilitators for Type B facilitation are BMcC and AT.

##### Criteria for selecting internal facilitators (for both Type A and Type B facilitation)

The following characteristics are discussed with the manager of the setting to select an appropriate internal facilitator:

1. Has some knowledge of good practice in continence care and has an interest in the topic

2. Knows co-workers

3. Knows the environment

4. Knows the organisation

5. Occupies a clinical leadership position

6. Possesses effective communication skills

7. Is self-aware and resilient

8. Is reliable and dependable

##### Recommendations (the evidence) being implemented

A review of research evidence was conducted as part of the fourth International Consultation on Incontinence (ICI) [43]. They based their recommendations on gradings derived from the Oxford Centre for Evidence Based Medicine 'Levels of Evidence' [44]. Grading for therapeutic interventions included Grade A: consistent evidence from meta-analysis of trials or good quality RCTs; Grade B: consistent evidence from lower quality RCTs, meta-analysis with homogeneity, good quality prospective cohort studies, good quality retrospective case controls studies, good quality case series; Grade C: expert opinion based on first principles or majority evidence from study types listed in Grade B, or Delphi processed expert opinion. Grade D: no recommendation is possible as evidence is conflicting or expert opinion has not been through a formal analytical process. They note that a Grade A recommendation often means the recommendation is effectively mandatory and placed within a clinical care pathway.

The recommendations to be implemented in this study were thus taken from the algorithm developed by Committee 11 who examined the research relevant to Incontinence in the Frail Elderly [43]. The drafted recommendations were then circulated to continence experts in each country and the continence experts on the study's Advisory Committee. No country-specific issues that may make their implementation problematic were identified by the continence experts. The recommendations [43] to be implemented in the FIRE study are listed below, with their associated level of evidence.

Recommendations to be implemented in FIRE study

1. The patient/resident should be actively screened for urinary incontinence.(Grade A)

2. A detailed assessment should be carried out including:

• Relevant co-morbid conditions should be assessed:

- rectal examination; constipation (Grade C)

- functional assessment (mobility, transfer, dexterity, ability to toilet) (Grade A)

- screen for depression (Grade B)

- assess cognitive status (Grade C)

- any medications that could cause or worsen UI (ICI recommendation)

- any medical conditions that could cause or worsen UI (ICI recommendation)

• Urinalysis should be undertaken (including for haematuria, leucocytes, nitrites) (Grade C)

• Undertake wet checks to assess frequency of urinary incontinence (Grade C)

• Ensure diagnosis of the type of UI is recorded (ICI recommendation):

- Urgency

- Stress

- Mixed

- Other

3. An individualised treatment plan should be in place for:

• Individualised goals of care (Grade C)

• Treatment preferences of resident and/or next of kin (Grade B)

• Bladder retraining (Grade A), or tailored prompted voiding [if able to state name] (Grade A)

• Degree of bother to resident/next of kin of UI (Grade B)

4. Specialist referral should be made if needed (pain, haematuria, UI not classified at urgency, stress or mixed). (ICI recommendation).

##### Ethical Issues

The researchers will adhere to ethical standards for research involving people. In addition, all researchers will abide by their institutional and national ethical requirements. Ethical Committee approval was obtained in England, Sweden, and Republic of Ireland. In the Netherlands, the researchers were advised to get permission from either an ethical committee at site level, or where this did not exist, from a scientific or residents committee at the site. Research Governance approval was also obtained in England, and permission to collect data at the sites obtained in Sweden and Republic of Ireland.

#### Outcomes

##### Primary outcome

Percentage compliance with continence recommendations (Additional file table S1) will be calculated, and a data profile constructed.

#### Secondary outcomes

##### Clinical outcomes

These include number of referrals for specialist continence assessment, incidence of documented incontinence-related dermatitis, urinary tract infections (documented as confirmed by laboratory, confirmed by dipstick or confirmed by symptoms only), and patients' health-related quality of life using I-QoL [45,46] and the EQ-5D [47,48]. The impact of length of stay will be investigated in the analysis, treating length of stay as a co-variant.

##### Process evaluation data

The embedded process evaluation will focus on four areas: facilitation residential programme evaluation; facilitation (i.e., intervention) implementation (i.e., how the intervention plays out in practice); influences on implementation (i.e., enablers and barriers); and the impact of intervention implementation on continence care processes and care home environments.

Qualitative and quantitative data collection methods will be used to strengthen our conclusions through method triangulation. Consistent with a realistic evaluation approach [20,49] our approach to data collection, analysis and integration will be guided by a number of propositions, developed in the initial stages of the study from our conceptual framework, which will be tested throughout the study.

##### Methods of data collection for process evaluation data

Data are being collected through interviews with relevant stakeholders including staff, residents, and next of kin, a questionnaire to assess the context of practice (Alberta Context Tool, ACT), observations and documentary evidence. Our intention is to construct a comprehensive picture of the impact of facilitation on continence practice, nursing home contexts, and residents.

##### The Alberta context tool

ACT has been designed to be a reliable and valid measure of context within complex healthcare settings where care is provided to patients/residents [29]. The ACT is a short questionnaire based on the construct of context (culture, leadership, evaluation) within the PARIHS framework. Additionally, it assesses a number of other concepts that have been found to be important in knowledge translation activity, including structural resources, social capital, organizational slack, and formal interactions. ACT has been designed to be completed by individual care providers, and a number of different versions have been developed for different disciplines and levels of nurses (see [29] for more details). The administration of this instrument will provide us with an assessment of the context in which care is being delivered within the study's nursing homes, and any factors that may explain implementation activity and outcome. Our target sample for the ACT is 30 staff in each home. All nursing staff (licensed practical nurses, registered nurses, and healthcare assistants) who plan and deliver direct care to residents, including continence care, will be asked to complete the questionnaire and return it directly to the research team and/or data entry company. Staff will be identified by the local link person, and the survey administered through the internal postal system. The survey will be administered on three occasions: at baseline, then 12 and 24 months after the intervention. Consent to participate in this part of the study will be assumed if the person decides to complete and return the questionnaire. A second distribution of the questionnaire with a letter reminding staff will be sent to all staff between three and six weeks after the first distribution.

##### Interviews

Semi-structured interviews (telephone and face-to-face) will be conducted with internal and external facilitators, staff, stakeholders, and residents as well as their next of kin during the lifetime of the project. Interviews will be conducted at critical points in the programme pre-, during, and post-intervention. Interviews will focus on the experience of implementation, barriers and facilitators, key events, social, political, and financial aspects of care and service delivery, and more specifically how continence care is delivered. The questions asked in interview will be adapted, depending on the purpose of the interview, and who the stakeholder is.

Data from interviews will enable the capture of intervention specific process information (i.e., about continence care) as well as more general features of the study sites with which to contextualise outcome data from the main intervention project. Interviews will be audio-recorded, and later transcribed.

##### Non-participant observation

We will undertake non-participant observation of care delivery, significant events in the implementation processes and facilitation residential programmes. Non-participant observation will contribute to identifying barriers and facilitators to implementation and practice change, to provide evidence about the role and influence of the facilitators including identifying what are the elements of the role that contribute to 'good enough' facilitation, the reasons for the particular adaptations of the facilitation models to local contexts, and the interactions between facilitation and the context(s) of care.

Observations will be recorded as field notes, including descriptive accounts, and interpretive/reflective comments. Where appropriate and possible, interviews will be timed to follow periods of non-participant observation. Observations will be informed by nine dimensions (space, actors, activities, objects, acts, events, time, goals, and feelings) [50] and the elements contained in the evaluation strands. Anyone who does not wish to be observed will not be part of the observation and no data will be collected relating to them.

##### Routinely collected data

We will gather any available routinely collected local data relevant to the delivery of continence care, and to help us understand the context of care. This may include, for example, an audit of continence care or product use.

##### Documentation

Relevant continence care specific documentation will be collected from each site such as policies, procedures, and guidelines. These should provide useful sources of information about expected continence care practice. Documents will be monitored through from pre- to post- intervention, and any changes made to them noted.

##### Intervention-specific process data

Process data will be collected by facilitators as part of both facilitation interventions, which may include for example the administration of the Context Assessment Index [51], internal facilitators' activity logs, any audits or other evaluations they or the nursing homes conduct during the project, data collected routinely as part of the facilitators' development programmes (e.g., portfolios or reflective diaries, supplies data, policy documents), data collected as a requirement of registration of external audit procedures (appropriate permissions to use these data in anonymised form will be sought).

##### Economic evaluation

The embedded economic evaluation will take a public sector, multi-agency perspective [52,53]. The analysis will be guided methodological best practice for conducting economic evaluations and cost analyses of guideline implementation strategies [54]. The interventions (i.e., facilitation models to enhance compliance with the recommendations) will be fully costed using the finance records plus internal facilitator diaries to record any additional costs that are not fully funded by the study. We will take care to distinguish between research costs and true intervention costs. These facilitation model costs will include residential schools, teleconferencing, travel, salaries, locum support, study time, email, and other support time. In order to find out the ongoing costs of managing continence at study sites, we will collect staff activity and incontinence product use information. Nationally agreed midpoint salaries for the staff grade will be attributed, as appropriate to each participating country. Interviewers will collect costs and types of incontinence products from residential site managers. The time horizon for the trial is two years. All costs will be reported in Euros for the year of study completion. Costs in year two will be discounted at appropriate rates. We will conduct a primary cost-effectiveness analysis to determine cost (in Euros) of achieving various levels of compliance (%) with the recommendations. We will conduct a secondary cost consequence analysis comparing the costs of different facilitation models with a full range of consequences. Amongst these consequences will be self- and proxy-reported health-related quality of life using EQ-5D [55]. Sensitivity analyses will be conducted to see how level of compliance with the recommendations varies according to our assumptions. In our cost effectiveness analysis, we will fully address uncertainty and use cost-effectiveness acceptability curves (CEACs) to convey to policy makers the probability that facilitation models are cost effective at different payer thresholds e.g., £20,000 to 30,000 used by NICE in UK. Subgroup analysis will consider important predictors that may affect quality of life during follow-up, including age, dementia status, or those referred for an incontinence assessment, presence of incontinence related dermatitis, and country of residence.

#### Sample size

##### Power calculation

There is no information available on the current level of compliance with the recommendations, so for the purposes of the power calculation our initial assumption is that there is 50% compliance. It is assumed that in each nursing home 50 patients would be available for assessing compliance with the recommendations. For 90% power to detect if compliance with the recommendations is 15% better in the facilitation arms compared to the standard dissemination arm and allowing for an intracluster correlation of 0.01 and statistical tests carried out at the 5% level, we will, for cluster size 50, require 7 clusters (nursing homes) per intervention arm [56]. Thus 21 clusters in all will be needed. Allowing for potential attrition, this is increased to 8 clusters per intervention arm, so 24 clusters in total. This equates to 6 long term nursing care settings per country with 50 patients per long term care setting.

If compliance with recommendations is lower than 50%, for example 10% (0.1) then for 50 residents per cluster, 4 clusters would be needed per intervention (total 12 clusters). If there were 20 residents per cluster, then 8 clusters per intervention would be needed (total 24), and if 10 residents per cluster then 14 clusters per intervention (total 42) would be needed.

This gives the range of number of clusters potentially needed, given lack of information on current compliance, and allowing for a range of numbers of available residents in each cluster. In this study, it was decided that having 8 clusters per intervention (total 24) would give the study the best chance of detecting a 15% improvement in compliance between the standard dissemination and facilitation arms.

##### Qualitative sample size

Up to five members of nursing staff, five residents, five next of kin and managers and other stakeholders will be interviewed in each site at baseline. Exact numbers will be determined by data saturation and local factors. Further interviews to explore the processes of implementation and its impact will be conducted at points during the study where there have been changes either to continence care or in the context locally at the home, region or nationally.

##### Randomisation

In each country (UK, Sweden, Netherlands, and Republic of Ireland), six sites will be selected which meet the inclusion criteria. These sites will be randomised from a central randomisation point, which will ensure allocation concealment. We will use a stratified random allocation, stratifying by country, and within each strata we will use randomised block of size six. Each site will be randomly allocated to one of three groups: standard dissemination; Type A (technical) facilitation; or Type B (enabling) facilitation. In each country there will be two sites randomly allocated to each arm. The randomisation schedule will be computer generated and prepared by a statistician who is independent of the project team. The country co-ordinators will convey allocation to each site to ensure the research fellows remain blinded.

##### Blinding

There are many challenges of maintaining blinding in this study. However, research fellows collecting outcome and process data will be blind to the random allocation of each site for baseline data collection. From previous experience, we anticipate that the blinding may be inadvertently broken by the sites either during or after baseline data collection. (For example, by referring to external facilitators by name). If and when blinding is broken will be recorded for each site. It is not possible to blind the external facilitators delivering the facilitation programmes as they are delivering the programme because they have specific expertise in that type of facilitation.

##### Timing of data collection

Baseline data will be collected at each site before the intervention. The intervention will take place over 12 months in arm two, and over 24 months in arm three. Outcome data will be collected at 6, 12, 18, and 24 months after the start of the intervention for all groups. This will be concurrent with longitudinal process evaluative data collection.

##### Translations

Interview schedules and recommendations were translated into Dutch and Swedish. These translations were checked by the country co-ordinators. The Quality of Life Scales already had validated Swedish and Dutch versions. The Alberta Context Tool was translated into Swedish and Dutch. A systematic process of translation, back translation, revision, and feasibility assessment based on several years experience and translation into several languages with the ACT was followed.

##### Research fellow training

Research Fellow training was provided before baseline data collection to ensure a common understanding of the process, and reinforced at training sessions throughout the study.

#### Analysis

##### Quantitative analysis

Data will initially be entered onto an excel spreadsheet, uploaded by each country onto a secure central site, and then transferred to SPSS version 18 for analysis. An intention to treat analysis will be carried out on the primary and secondary outcomes. Descriptive statistics will be calculated for all variables of interest. Continuous measures will be summarised using means and standard deviations; categorical variables will be summarized using counts and percentages. Descriptive analyses within and across data sets and within and across sites by country, as well as across countries, will be carried out.

The primary outcome measure, compliance with the recommendations, will be compared at cluster level [56]. Repeated measures analysis of variance will be used to investigate the change in compliance over time by intervention group. Analysis of subgroups and of changes between specific time points can be explored with t-tests or analysis of variance and where appropriate multilevel hierarchical modelling will be used. However, the study is not expected to be sufficiently powered for detailed subgroup analysis.

A Data Management and Monitoring Committee has been set up with agreed terms of reference to review quantitative data management and monitor emerging results.

##### Qualitative analysis

Each set of data will first be analysed separately first using a thematic content analysis, within a realist evaluation framework [40]. This focuses the analysis on developing and testing theories of what works, for whom, how, and in what circumstances by identifying outcomes, and the contexts and the mechanisms by which the outcomes are achieved. The data sets will then be synthesised drawing on the principles of realistic evaluation. We anticipate that it may be possible to generate some mid-range theory from this data analysis that will contribute to better understanding 'good enough' models of facilitation for implementing guideline recommendations and the influences of context on the processes of implementation.

Qualitative data will managed through AtlasTi. Data analysis will be an iterative process between data sets and study phases. Integration and synthesis of data will be guided by our theoretical framework and principles of conducting mixed methods research [57].

##### Knowledge translation strategy

An integrated knowledge transfer strategy underpins the study, encompassing three key strands of activity. First, we are developing and implementing a model of stakeholder involvement that complements the theory-driven approach to the study design and enables stakeholder involvement throughout the research process. Second, we are developing a portfolio of networking and dissemination activities that will promote active diffusion of the study findings to key individuals and organisations in the EU, Europe and internationally. Third, we will be supporting study participants to disseminate their experiences and learning as a result of their involvement in the research, thus enhancing the sustainability and spread of the study findings. We will evaluate the effectiveness of stakeholder involvement and dissemination activities as the study progresses and revise accordingly.

##### Study governance

The Committee and Management Structure include:

1. A project board comprising all members of the consortium, chaired by project co-ordinator.

The Project Board assures co-ordination and supervision of all activities, including time lines and project reports and publications. The Board will meet twice a year, and maintain regular communication as appropriate. Decisions will be normally taken by consensus. In exceptional cases where this is not possible a majority vote will be used.

2. An advisory committee comprising key stakeholders and including external experts, to provide advice and review progress.

Their role is to provide advice, guidance, and challenge to the project board throughout the project, and to help ensure the research contributes to knowledge at a European and International level, but final responsibility lies with the project board. This committee will meet three times, at the start, mid- and end-phases of the study (6, 24, and 42 months).

##### Management of study

KS is the overall project co-ordinator. Each country site will be managed and overseen by a member of the consortium nominated as country co-ordinator. (Sweden LW; Republic of Ireland BMcC; Netherlands KC; UK JRM).

## Discussion

Initial experiences with data collection so far suggest this pan-European study is complex to run, with many and varied challenges. A 'lessons learned' log and risk register are important aspects of managing these challenges.

New understandings and empirical testing of the facilitation element of the PARIHS framework have the potential to make substantial contributions to knowledge in this area.

## Competing interests

The authors declare that they have no competing interests.

## Authors' contributions

KS (principal investigator) led the application for funding. She contributed to the overall design of the study and designed to pragmatic RCT aspect of the study. She led the design of the outcomes to be assessed and led the writing of this protocol manuscript. KC (collaborator) participated in designing the study and provided critical commentary to the manuscript. She is country co-ordinator for the Netherlands. NC (statistician) advised on study design and contributed to the analysis plan within the pragmatic RCT, and provided critical commentary on the manuscript. RTE (health economist) is responsible for the economic evaluation study design and has contributed to the manuscript. ACE (research fellow) participated in the design and development of the evaluation package and commented on the manuscript. CAE (collaborator) participated in study design, participates in the evaluation stream and coordinated the use of the Alberta context tool including its translation into Swedish and Dutch. She commented on the manuscript. GH (collaborator) participated in the design of the overall study and in the design of the facilitator intervention in particular. She co-leads Arm two of the intervention and work package that is concerned with knowledge translation and dissemination. GH has inputted to and commented on the manuscript. AK (collaborator) participated in the design of the overall study and in the design of the facilitator intervention in particular. She co-leads Arm two of the intervention. AK has commented on the manuscript. PL (health economist) contributed to the economic evaluation design, is responsible for the day to day running of the economic evaluation, and contributed to the manuscript. BMcC (collaborator) participated in the design of the overall study and in the design of the facilitation intervention in particular. He contributed to the text about Arm three and commented on drafts of the manuscript. He co-leads Arm three of the intervention and is the Country Coordinator for Ireland. GMcC (collaborator) participated in the initial design of the study and commented on the manuscript. CH (research fellow) participated the design of the process evaluation and associated data collection tools, the development of the economic evaluation, and is responsible for the day to day running of the process evaluation. She commented on the manuscript. CM (research fellow) participated in the design of the RCT and associated data collection tools. She commented on the manuscript. JRM (collaborator) participated in designing the study. She led the design of the evaluation package and is country co-ordinator for England. JRM commented on drafts of the manuscript. AT (collaborator) participated in the design of the overall study, especially the Type B facilitation intervention and the design of the evaluation package. She co-leads Arm three of the intervention, has contributed to the text about Arm three and commented on the manuscript. LW (collaborator) participated in the design of the overall study and in the design of the intervention evaluation in particular. He has commented on the manuscript. He is the Country Coordinator for Sweden. All authors read and approved the final manuscript.
